# DCTR U-Net: automatic segmentation algorithm for medical images of nasopharyngeal cancer in the context of deep learning

**DOI:** 10.3389/fonc.2023.1190075

**Published:** 2023-06-30

**Authors:** Yan Zeng, PengHui Zeng, ShaoDong Shen, Wei Liang, Jun Li, Zhe Zhao, Kun Zhang, Chong Shen

**Affiliations:** ^1^ State Key Laboratory of Marine Resource Utilization in South China Sea, School of Information and Communication Engineering, Hainan University, Haikou, China; ^2^ ChinaPersonnel Department, Hainan Medical University, Haikou, China; ^3^ School of Information Science and Technology, Hainan Normal University, Haikou, China

**Keywords:** deep learning, nasopharyngeal carcinoma, automatic segmentation algorithm, dilated convolution, transformer module, residual module

## Abstract

Nasopharyngeal carcinoma (NPC) is a malignant tumor that occurs in the wall of the nasopharyngeal cavity and is prevalent in Southern China, Southeast Asia, North Africa, and the Middle East. According to studies, NPC is one of the most common malignant tumors in Hainan, China, and it has the highest incidence rate among otorhinolaryngological malignancies. We proposed a new deep learning network model to improve the segmentation accuracy of the target region of nasopharyngeal cancer. Our model is based on the U-Net-based network, to which we add Dilated Convolution Module, Transformer Module, and Residual Module. The new deep learning network model can effectively solve the problem of restricted convolutional fields of perception and achieve global and local multi-scale feature fusion. In our experiments, the proposed network was trained and validated using 10-fold cross-validation based on the records of 300 clinical patients. The results of our network were evaluated using the dice similarity coefficient (DSC) and the average symmetric surface distance (ASSD). The DSC and ASSD values are 0.852 and 0.544 mm, respectively. With the effective combination of the Dilated Convolution Module, Transformer Module, and Residual Module, we significantly improved the segmentation performance of the target region of the NPC.

## 1 Introduction

One of the primary pillars of contemporary medical diagnosis is the medical image, which is an image that depicts the interior organization of the human body. At present, medical image segmentation mainly focuses on photographs of diverse human organs, tissues, and cells. The goal of medical image segmentation is to separate the image into several areas based on how similar or dissimilar the regions are to one another. Researchers have been investigating and proposing numerous technologies and approaches to medical image segmentation over the past several years, and many of these methods have been successful in image segmentation. The performance of the technique based on standard machine learning is rather constrained since the capacity of extracted features to express themselves is constrained due to the method’s primary reliance on feature engineering. Deep learning techniques, particularly those based on convolution neural networks, have recently been more effective than classic machine learning techniques in a variety of applications, including the segmentation of medical images. Deep learning-based medical image segmentation techniques are getting more and more attention (1).

Medical images, including magnetic resonance imaging (MRI) and electronic computed tomography (CT), are non-invasive and can help in the diagnosis of malignant lesions. They are often employed in the auxiliary diagnosis of new coronavirus (2019-nCov) and are crucial in controlling the pandemic of novel coronavirus (2). Because MR images provide a high spatial resolution for soft tissue, it is frequently employed in the clinical diagnosis of nasopharyngeal cancer. For the assessment of radiation and follow-up treatment of nasopharyngeal carcinoma (NPC), the localization of NPC lesions using MR images is of tremendous reference relevance. Currently, radiologists must perform a physical inspection to identify and confirm nasopharyngeal cancer lesions in MR images. However, the MR images were created by layer-by-layer scanning and are three-dimensional. Consequently, the layer-by-layer evaluation of the MR images by the imaging physician is a tedious task that is readily hampered by visual fatigue and accuracy (3). Additionally, the clinical expertise and professional competence of imaging experts have a role in the accuracy of manual inspection. Doctors who specialize in imaging will misdiagnose nasopharyngeal cancer due to their lack of clinical experience and technical skills. The information shows that China’s average misdiagnosis rate for NPC and other cancerous tumors has surpassed 40%.

The most common cancer among ear, nose, and throat tumors, with a high frequency in Hainan, China, is nasopharyngeal cancer, which frequently develops in the epiglottis and nasopharyngeal cavities. Owing to the high sensitivity of nasopharyngeal cancer to radiation, radiation therapy has become the preferred treatment option for irradiating nasopharyngeal cancer tumors. Compared to conventional nasopharyngeal cancer treatment methods, radiation therapy has increased patients’ 5-year survival rates to 70% (4). If the specific position of the target region of the NPC is properly segmented before radiation therapy of NPC tumors, the effectiveness of the procedure and the extent of post-operative recovery will be considerably increased.

Experienced radiotherapists found that due to the diverse morphology of the disease and the significant number of nearby organs at risk, partitioning manually the nasopharyngeal cancer target region and organs is challenging and time-consuming and risky. Additionally, segmenting the target region and organs at risk manually is quite subjective, and various radiotherapists have varied criteria and methods for segmentation. With the development of computer science and technology, medical image analysis and computer aid have been extensively employed in clinical applications, such as medical image reconstruction, alignment, classification, segmentation, and other domains. Medical image segmentation is the segmentation of organs, lesions, and tumors, and further evaluation and treatment of diseases according to the results of image segmentation. Usage of computer-assisted automatic segmentation of target regions and organs at risk in nasopharyngeal cancer can standardize segmentation standards of medical images of nasopharyngeal cancer (5), reduce the risks of radiotherapy due to radiotherapists’ inexperience, and significantly improve the efficiency of radiotherapy planning for nasopharyngeal cancer patients, which makes this study clinically significant.

## 2 Related works

### 2.1 Traditional segmentation model

In earlier studies, traditional segmentation methods such as template matching, radio frequency, contouring, region growing, and support vector machine (SVM) had significant advantages over traditional manual segmentation. Xu et al. (6) proposed an SVM-based method for segmenting cancer regions by MRI. Mapping hyperplanes by kernel mapping allows them to perform the task automatically without *a priori* knowledge. Kass et al. (7) introduced a contour-based segmentation method that requires the physician to first outline a rough contour. Because of the underlying image features, their “snake” algorithm will adjust the rough contour to obtain more accurate results. In (8), a method was proposed that the user should first select an initial seed within the tumor, then based on the information obtained from the contrast enhancement ratio, the seeds grow to segment the NPC lesion in MRI. However, these methods are mainly based on the underlying features and are time-consuming.

### 2.2 Medical image segmentation in the context of deep learning

#### 2.2.1 Convolutional neural networks model for medical image segmentation

In recent years, convolutional neural networks (CNNs) have been intensively studied and explored by many experts and scholars because of their excellent performance, and they have been more and more widely used in the field of medical image segmentation. With the development of deep learning, fully convolutional networks (FCNs) (9), U-Net (10), DeepMedic (11), nnU-Net (12), and other CNN models have obtained high-quality segmentation results. Compared with traditional machine learning methods, CNN models can automatically learn different levels of feature structures directly from the original input image data without manually extracting and selecting useful features for classification. Several experts and scholars have applied CNN to segment medical tissue structures such as retinal blood vessels, pancreas, knee cartilage, and brain.

#### 2.2.2 Transformer model for medical image segmentation

In the field of medical image segmentation, the model based on CNNs has achieved good results. However, as the medical images have the characteristics of complex structure, difficulty in data annotation, and limited labeled data, it still has shortcomings such as insufficient segmentation accuracy and segmentation precision. Therefore, some scholars proposed applying the Transformer Model to ImageNet classification tasks in computer vision. Based on the use of multi-layer self-attention and multilayer perceptron, the Transformer Model establishes a sequence–sequence prediction method and models from an overall perspective. It has achieved great success in the field of natural language processing and image processing. The Transformer Model divides the image into fixed-size image patches and uses linear projection to project the patch flat onto a specified dimension to obtain a token sequence. The sequence is used as input for features, achieving a new segmentation mode. The Transformer Model achieves global information modeling without using down-sampling, ensuring image resolution is not reduced, which is a new semantic segmentation mode. Without the help of the hole convolution and FPN structures in the convolution method, the Transformer Model expands the receptive field and obtains the characteristic response from the global perspective.

The Swin-Net model has achieved good results in liver image segmentation by using a pure Transformer U-shaped network architecture (13). Because the calculation of the core self-attention in the Transformer is at the square level of image resolution, the number of parameters required for a Transformer calculation on a larger image is relatively large. Therefore, a sliding window operation is required to divide the entire feature map into several windows, with each window containing a fixed number of seven patches. Only the self-attention of patches within the window range is calculated each time. This reduces the computational time complexity. The Shift Windows method uses a loop to move the upper left corner, allowing non-adjacent patches within the window to communicate with each other. However, it has disadvantages in that the performance of the Transformer’s structure is relatively poorer on medical image datasets with limited data volume. The TransUNet model first used a U-shaped lightweight network that combines Transformer and CNN for abdominal organ segmentation (14). Low-level information is extracted by using conventional CNN. At the end of the encoder stage, the feature graph obtained by convolution will be serialized through the patch to get tokens. Finally, it obtains global information through Transformer, which achieves good segmentation effects.

#### 2.2.3 NPC segmentation based on deep learning

Men et al. (5) developed an end-to-end deep deconvolutional neural network (DDNN) for NPC segmentation. DDNN has the potential to improve the consistency of contouring and streamline radiotherapy workflows, but careful human review and a considerable amount of editing will be required. Ma et al. (15) proposed an automated multi-modality segmentation framework for NPC radiotherapy based on deep CNNs, which is the first CNN-based method that solves the problem of multi-modality tumor segmentation in the field of NPC. Guo et al. (16) proposed a GTV segmentation framework for HNC radiotherapy. This method employs 3D convolutions to take full advantage of 3D spatial information of images as well as dense connections to improve information propagation from multi-modality images. Lin et al. (17) proposed a deep 3D CNN to construct an AI contouring tool to automate gross tumor volume (GTV) contouring for NPC. AI assistance can effectively improve contouring accuracy and reduce intra-observer and inter-observer variation and contouring time, which could have a positive impact on tumor control and patient survival. Chen et al. (18) proposed a novel multi-modality MRI fusion network (MMFNet) to segment NPC based on three modalities of MRI. The MMFNet can well segment NPC with a high accuracy and the utilization of multi-modality MRI is meaningful for the segmentation of NPC. Li et al. (19) proposed a three-dimensional densely connected CNN with multi-scale feature pyramids (DDNet) for NPC segmentation. By adding feature pyramids, densely connected convolutional blocks can be adjusted into a new structure. The concatenated pyramid feature carries multi-scale and hierarchical semantic information, which is effective for segmenting different sizes of tumors and perceiving hierarchical context information. Mei et al. (20) proposed a 2.5D CNN for nasopharyngeal cancer target region segmentation, combined with a spatial attention mechanism, and won second place in the MICCAI StructSeg 2019 nasopharyngeal cancer target region segmentation challenge. Tang et al. (21) propose a Dual Attention-based Dense SU-net (DA-DSUnet) framework for automatic NPC segmentation. It is an encoder–decoder network taking 2D NPC MRI slices as input and outputting the corresponding segmentation results.

However, owing to the lack of labels in real environments, the practicality of fully supervised methods is greatly reduced. In contrast, semi-supervised methods have gained attention due to their exploration of inherent information in unlabeled data. Hu et al. (22) proposed a two-stage semi-supervised method for NPC segmentation from CT images. Extensive experiments demonstrate the effectiveness of our method in leveraging both the scarce labeled data and adequate unlabeled data and also show the great generalization capability on other segmentation tasks. Luo et al. (23) proposed a novel framework with Uncertainty Rectified Pyramid Consistency (URPC) regularization for semi-supervised NPC GTV segmentation. Concretely, they extend a backbone segmentation network to produce pyramid predictions at different scales. The pyramid predictions network (PPNet) is supervised by the ground truth of labeled images and a multi-scale consistency loss for unlabeled images. The method largely improved the segmentation performance by leveraging the unlabeled images, and it also outperformed state-of-the-art semi-supervised segmentation methods. Li et al. (24) presented an end-to-end model named NPCNet that offers a fully automatic tool to segment primary NPC tumors and NPC MLNs jointly. The effectiveness of NPCNet is demonstrated by extensive experiments on a large dataset. Three carefully designed modules, named PEM, SEM, and BEM, are integrated into NPCNet to overcome three main challenges: the variable locations, variable sizes, and irregular boundaries of tumors and MLNs. Tang et al. (25) proposed an uncertainty-guided network referred to as UG-Net for automatic medical image segmentation. Different from previous methods, the UG-Net can learn from and contend with uncertainty by itself in an end-to-end manner. Liao et al. (26) proposed a novel SSL-based deep learning model to delineate GTVnx and GTVnd in NPC. Different from previous fully SL-based models, the SSL-based model was capable of using limited labeled data to learn efficiently. Huang et al. (27) proposed a segmentation method for NPC based on dynamic PET-CT image data. This method uses a generative adversarial network with a modified UNet integrated with a Transformer as the generator (TG-Net) to achieve automatic segmentation of NPC on combined CT-PET images. Luo et al. (28) proposed an augmentation-invariant framework. The framework could boost the generalization and robustness of the DL model. Using the proposed framework and a mixed training set for network training produced more accurate segmentation results of GTV for both the internal and the external testing cohorts. The proposed framework is a potential solution for accurate and generalizable GTV delineation of NPC from multiple hospitals’ MRI images. As shown in **Table 1**, the quantitative comparison was conducted on NPC segmentation using different methods. The full name of TCIA is The Cancer Imaging Archive, funded by the Cancer Imaging Program (CIP) under the National Cancer Institute (NCI) in the United States. The full name of MICCAI 2019 is MICCAI 2019 StructSeg challenge (GTV segmentation task).

**Table 1:** Quantitative comparison of NPC segmentation results using different methods.

Proposed	Year	Method	Image used	Dataset	DSC (%)	ASSD (mm)
Men et al.	2017	DDNN	CT	In-house	80.9	–
Ma et al.	2018	CNN	CT and MRI	In-house	75.2	1.062
Guo et al.	2019	CNN	PET and CT	TCIA	73	–
Lin et al.	2019	3D CNN	MRI	In-house	79	2
Chen et al.	2020	MMFNet	MRI	In-house	72.38	2.07
Li et al.	2021	DDNet	MRI	In-house	72.1	1.399
Mei et al.	2021	2.5D CNN	CT	MICCAI 2019	65.66	3.98
Tang et al.	2021	DA-DSUnet	MRI	In-house	80.50	0.8021
Hu et al.	2021	CNN	CT	In-house	78.09	–
Luo et al.	2021	PPNet	MRI	In-house	82.64	1.48
Li et al.	2022	NPCNet	MRI	In-house	73.5	1.74
Tang et al.	2022	UG-Net	CT	In-house	81.58	1.0471
Liao et al.	2022	CNN	MRI	In-house	83	1.48
Huang et al.	2022	TG-Net	PET and CT	In-house	91.35	–
Luo et al.	2023	CNN	MRI	In-house	88	0.97

Asterisks indicate that the difference between the different methods is statistically significant using a paired t-test on DSC (p< 0.05).

The symbol “–” represents that there is no relevant indicator data in the method.

### 2.3 Our contribution

In this study, we propose a new deep learning network model, Dilated Convolution Transformer Residual (DCTR U-Net), based on the U-Net network, combining Dilated Convolution Module, Transformer Module, and Residual Module. This network model has better segmentation performance, which can make the whole segmentation process more accurate and stable. The specific research contributions are as follows.

We developed a new residual network module for our deep learning network architecture that combines Dilated Convolution Module and Transformer Module. Using the encoder–decoder structure of U-Net as the basic framework, we propose a novel deep learning network model Dilated Convolution Transformer Residual (DCTR U-Net) for further improvement to enhance segmentation performance. We used medical image data of 300 patients for training and validation of the overall network model and obtained excellent segmentation results.

## 3 Methods

### 3.1 Dilated convolution layer

During the training of the CNN, the feature map is generated by extracting the feature information of the input image by convolutional extraction, and then the feature map is subsampled. While reducing the dimension of the feature map, the number of receptive fields and channels of the feature map should be increased. However, image segmentation prediction is the output of pixels. Although the feature map of small size can be restored to the original image size after up-sampling, the spatial resolution of the feature map will be reduced and important feature information will be lost during down-sampling, which will lead to the loss of spatial hierarchical information, and the lost feature information cannot be recovered through up-sampling. Some existing studies perform multi-scale extraction by convolution kernels of different sizes or dilated convolution to complement feature information and enhance the perceptual field (29). Dilated Convolution aims to solve the problem of image resolution reduction and information loss due to down-sampling in the image semantic segmentation problem. Dilated Convolution introduces a dilation rate parameter compared with Standard Convolutional, and the dilation rate can control the distance between adjacent elements in the convolutional kernel, thus controlling the size of the convolutional kernel’s perceptual field, so that a convolutional kernel of the same size obtains a larger perceptual field (30). Convolution with holes injects holes into the standard Convolution Map to increase the receptive field and capture multi-scale contextual information. The actual convolution kernel size of Convolution with holes *D* is as follows:


(1) D=K+(K−1)(R−1)

**Figure 1:**
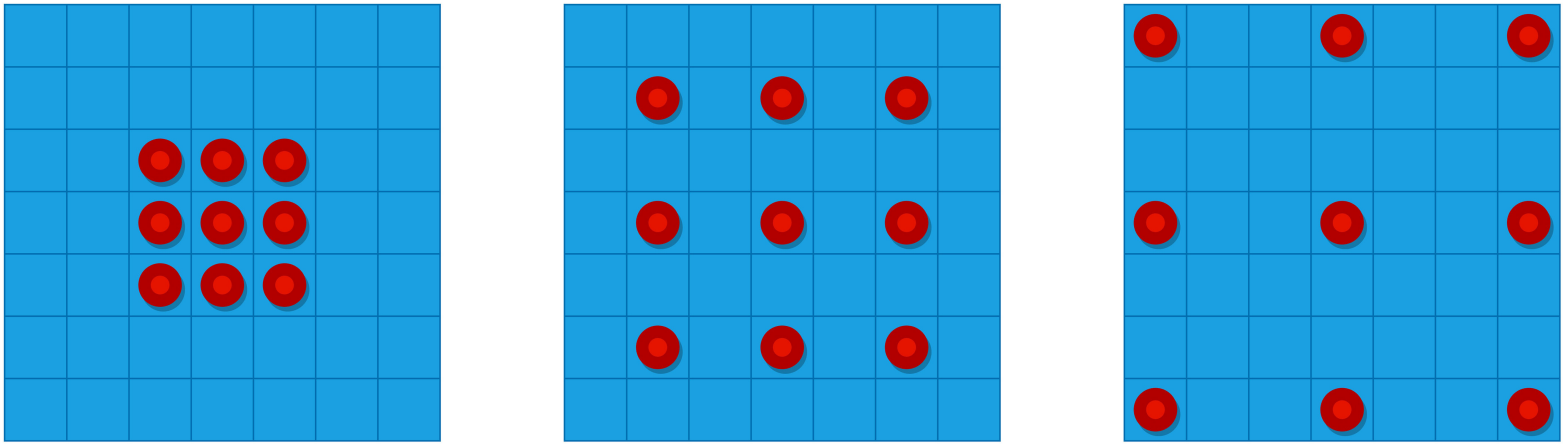
Dilated convolution with expansion rates of 1, 2, and 3.

*K* is the original convolution kernel size. *R* (Dilation Rate) is the Dilated Convolution expansion rate and the standard convolution *R* = 1. When *R* = 2, the 3 × 3 convolution kernel size is expanded to 5 × 5. When *R* = 3, the convolution kernel size is expanded to 7×7. For the same receptive field size, the hollow convolution has fewer numbers of parameters than the normal convolution. The Dilated Convolution with expansion rates of 1, 2, and 3 is shown in **Figure 1**, and the perceptual fields after convolution are 3, 5, and 7, respectively. The dilated convolution increases the perceptual field, so that each convolution output contains a larger range of information, thus supplementing the lost information caused by down-sampling, obtaining multi-scale feature information, and improving the accuracy of semantic segmentation.

### 3.2 Transformer layer

**Figure 2:**
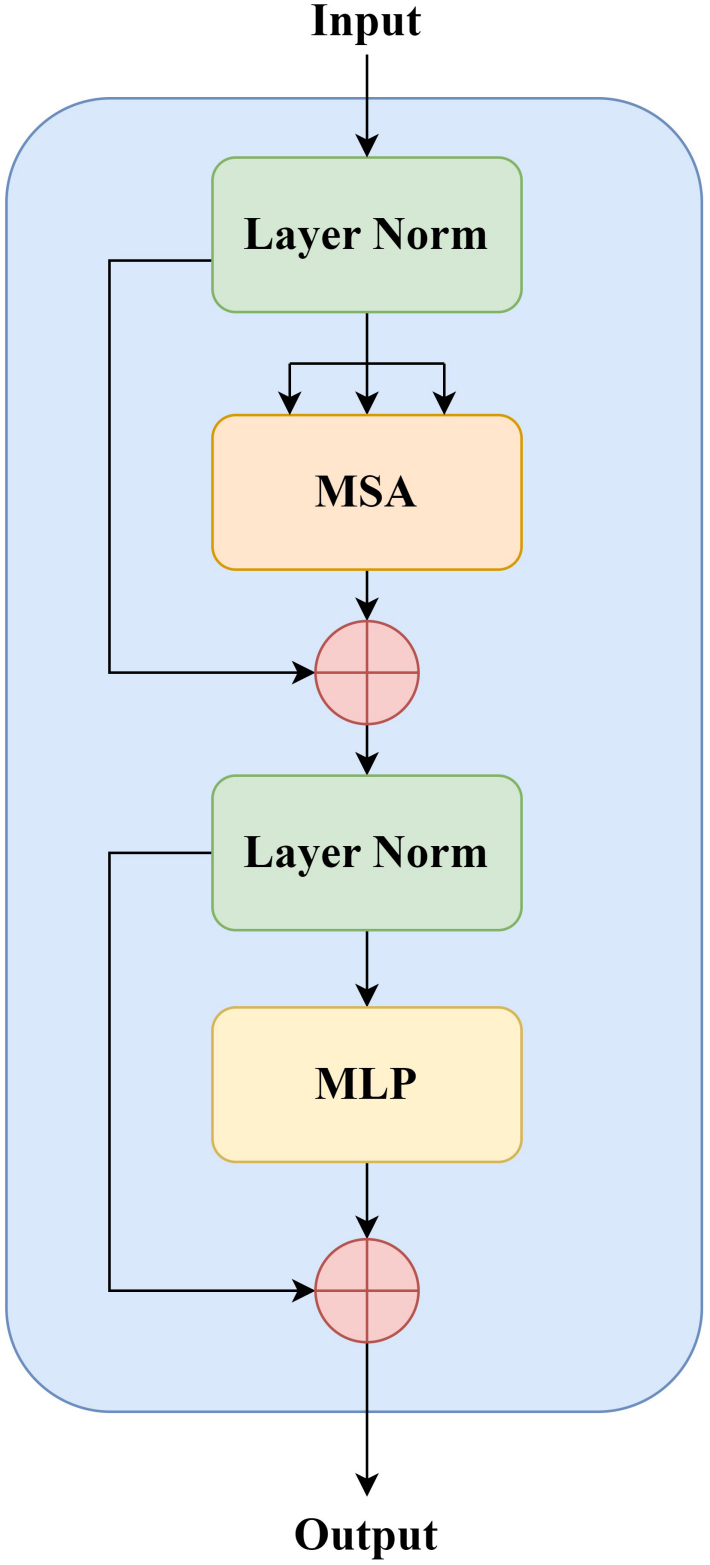
Transformer block.


The implementation of the standardized Transformer Block is shown in **Figure 2**, which contains two basic units, MSA (Multi-head Self-Attention) and MLP (Multilayer Perceptron). Before each MSA and MLP operation, normalize the data through the LN (Layer Normalization). Assuming that the input tokens at layer *l* is *x_l−_*_1_, then the calculation process of layer output obtained by the Transformer standard module is shown in publicity.

(2) $$\{\begin{array}{l} x_{l}^{'}=MSA\left(LN\left(x_{l-1}\right)\right)+x_{l-1}\\ x_{l}=MLP\left(LN\left(x_{l}{\;}^{\prime }\right)\right)+x_{l}^{'} \end{array}$$

In recent years, based on the powerful global contextual information representation capability of the Transformer Model, researchers have devoted themselves to applying the Transformer Model to medical image segmentation in the target region of NPC. Although the Transformer Model can establish global context dependence, it can destroy the shallow features of the convolutional network, and the local information contained in these underlying features is of great importance to improve the accuracy of edge segmentation. Therefore, designing more suitable CNN and Transformer fusion models that can establish good long-term dependency while preserving low-level information is a key problem to be solved.

We proposed a combination of Transformer and CNN to achieve the fusion of global and local features. On the basis of retaining the encoder–decoder, the loss of global information is compensated by introducing the Transformer Block in the last layer of the Encoder part, which effectively improves the segmentation accuracy compared with the pure Transformer approach. The equations should be inserted in editable format from the equation editor.

### 3.3 DCTR block

**Figure 3:**
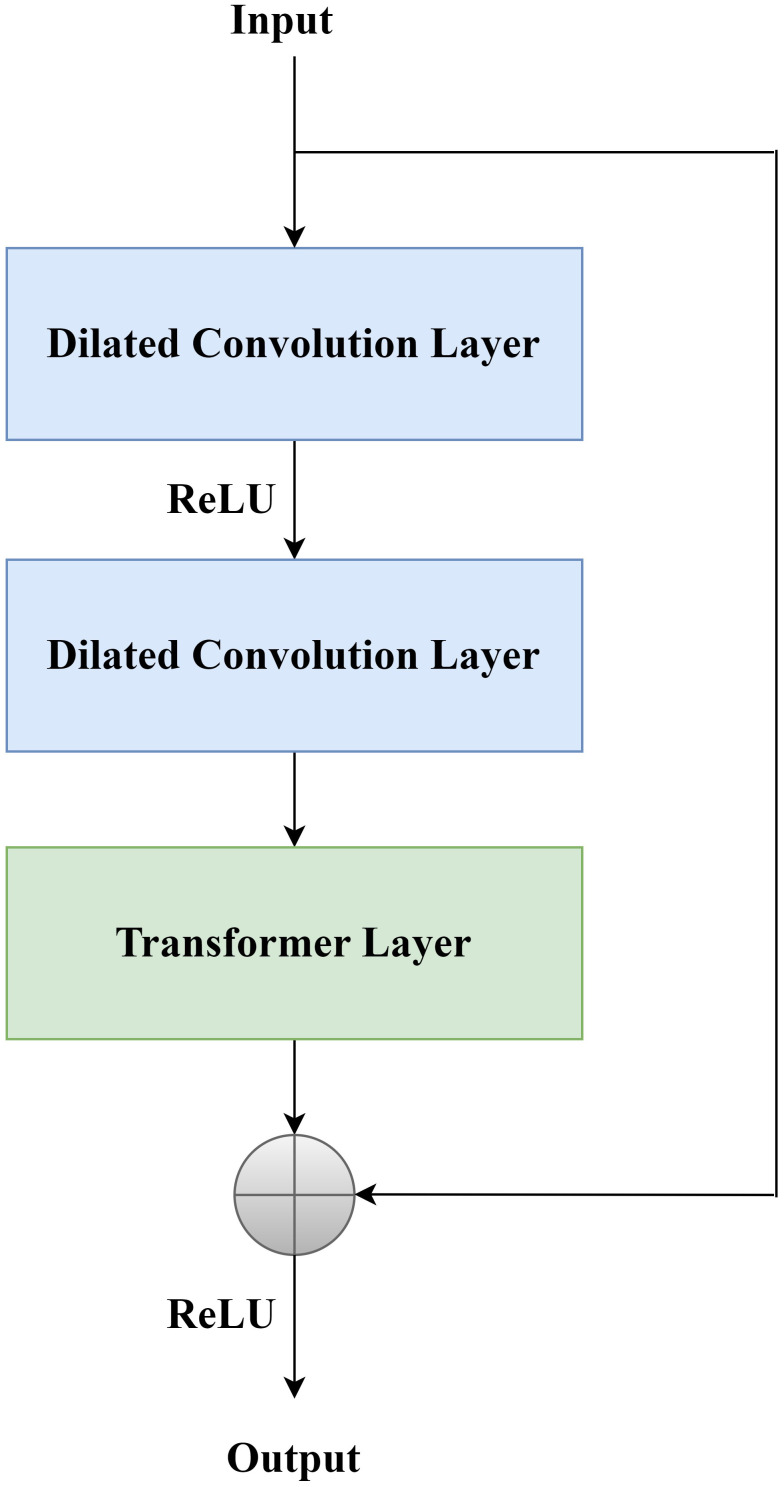
DCTR block.

The function of the ReLU activation function is to increase the nonlinear relationship between different layers of the neural network. Otherwise, if there is no ReLU activation function, there is a simple linear relationship between different layers, and each layer is equivalent to matrix multiplication, which cannot complete the complex task of our neural network (31). The ReLU activation function is defined as follows.


(4) $$y=\{\begin{array}{ll} 0, & x<0\\ x, & x\geq 0 \end{array}$$

where *x* is the input and *y* is the output. The DCTR Block structure is shown in **Figure 3**: after coming in from the input, it is first divided into two parts and passed in parallel. The first part of the input passes through the dilated convolution layer and then performs ReLU activation, followed by passing through the dilated convolution layer again, and then passing through the Transformer layer, and the second part of the input is summed with the first part of the input and passed through ReLU activation for output.

#### 3.3.1 Dilated residual module

The structure of the Dilated Residual Module is as follows: after coming in from the input, it is first divided into two parts and passed in parallel. The first part of the input is passed through the dilated convolution layer for ReLU activation, followed by another pass through the dilated convolution layer for ReLU activation, and the second part of the input is summed with the first part of the input for output.

#### 3.3.2 Residual transformer module

The structure of the Residual Transformer Module is as follows: After the input comes in, it is first divided into two parts and passed in parallel. The first part of the input passes through a 3×3 convolutional layer and then undergoes ReLU activation, followed by another 3×3 convolutional layer and then passes through the Transformer layer, and the second part of the input is summed with the first part of the input and then undergoes ReLU activation The output is performed.

### 3.4 DCTR U-Net

**Figure 4:**
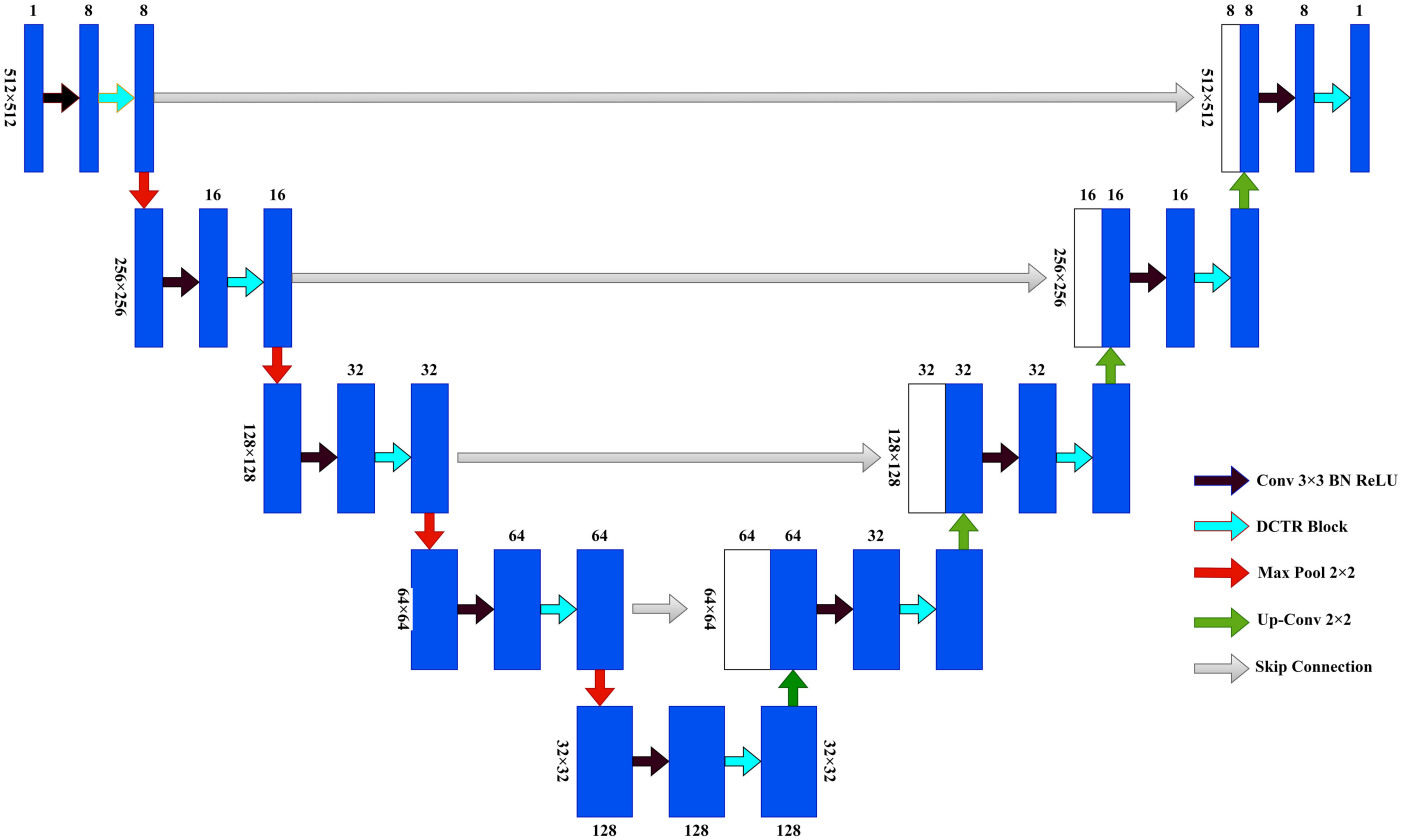
DCTR U-Net.

To effectively solve the problem of restricted convolutional field and to achieve global and local multi-scale feature fusion, the study proposes a new network model, Dilated Convolution Transformer Residual U-Net (DCTR U-Net), based on the U-Net model, combining the dilated convolution, transformer, and residual modules (32). The structure of the DCTR U-Net is shown in **Figure 4**.

The overall architecture of DCTR U-Net is divided into two parts, encoder and decoder. The encoder part consists of four down-sampling modules and a bottom connection module, for MR images of nasopharyngeal cancer with a size of 512×512×1. After the input of the down-sampling module, it first passes through the 3×3 convolutional layer, followed by the BN layer and the ReLU layer, when the number of channels changes, then passes through the DCTR Block, when the number of channels remains unchanged, and finally passes through the maximum pooling layer. Each time after passing the DCTR Block, skip connections to the decoder is required to complete the connection operation between the encoder and the decoder. The operation of connecting by skipping layers can effectively reduce the loss of spatial information brought by the down-sampling process, and make the feature map recovered by up-sampling contain more low-level semantic information, which makes the experimental results more stable and accurate. The input of the bottom connection module is firstly passed through 3×3 convolutional layers, followed by BN and ReLU layers, and then through DCTR Block, and the number of channels is all unchanged (33).

The decoder part consists of four up-sampling modules and one bottom connection module. The bottom connection module of the decoder is the same as the bottom connection module of the encoder. After the input of the up-sampling module, it first passes through the up-sampling layer, at which time the number of channels is halved, then it is superimposed with the input of the encoder skip connections and restored to the original number of channels, and then it passes through the 3×3 convolution layer, followed by the BN layer and ReLU layer, at which time the number of channels is halved, and then it passes through the DCTR Block, at which time the number of channels remains unchanged. The final output MR image size of nasopharyngeal cancer is 512×512×1.

## 4 Experiment

**Figure 5:**
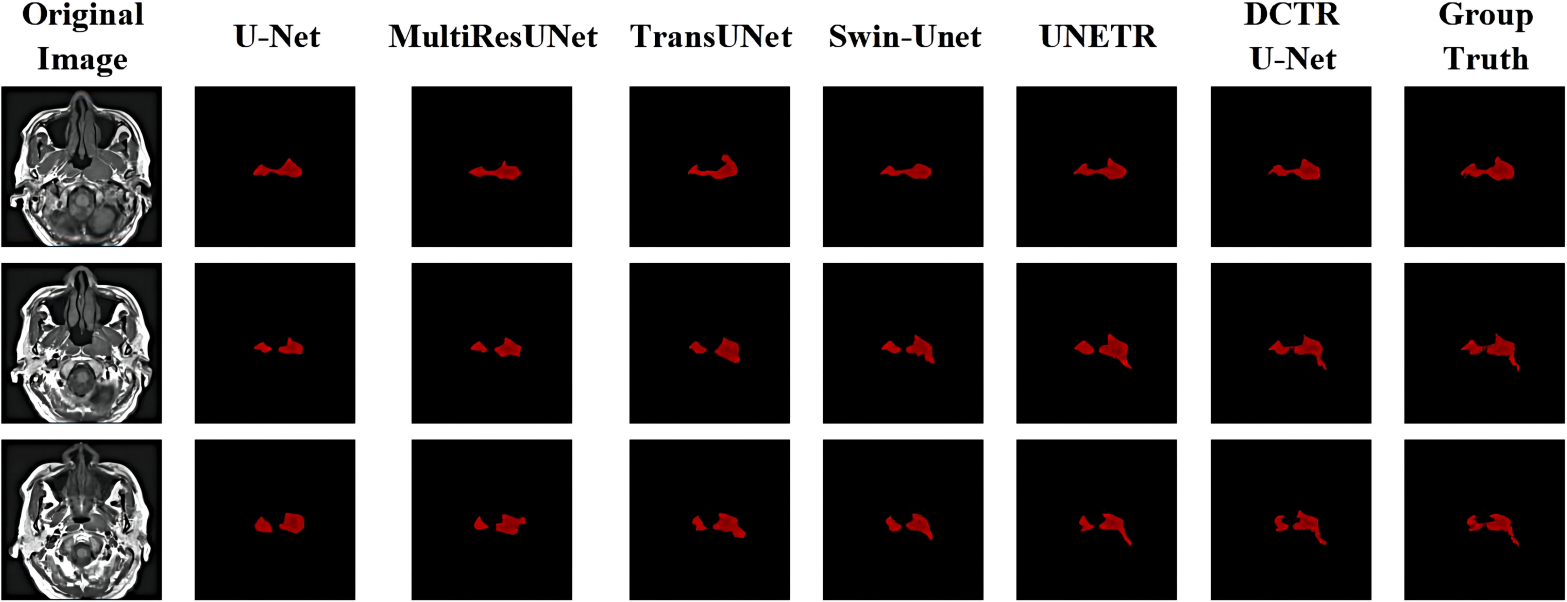
Visualization and qualitative comparison results under mainstream segmentation models.

**Figure 5** shows the qualitative comparison results under different segmentation models. For each subplot, the first column represents the original image, the second column represents the segmentation results under the U-Net model, the third column represents the segmentation results under the MultiResUNet model, the fourth column represents the segmentation results under the TransUNet model, the fifth column represents the segmentation results under the Swin-Unet model, the sixth column represents the segmentation results under the UNETR model, and the seventh column represents Ground Truth.

**Figure 6:**
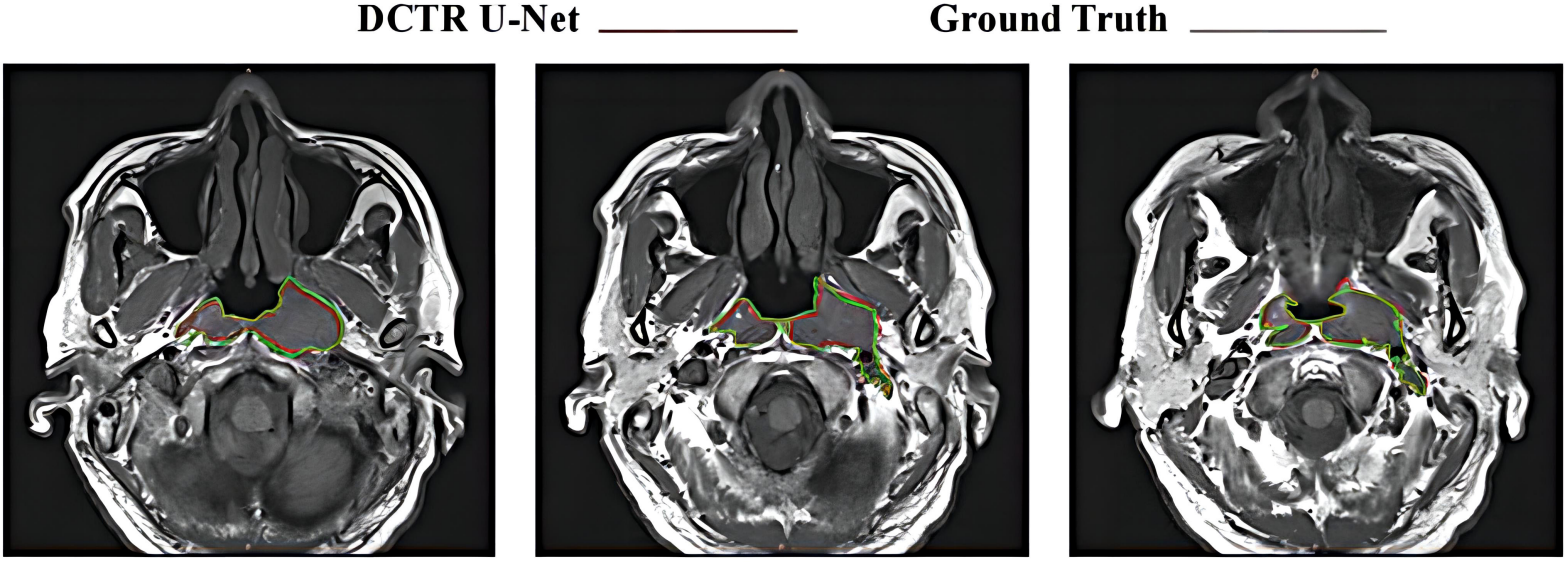
Differences between tumor contours generated by DCTR U-Net and Ground Truth.

**Figure 6** shows the consistency level of NPC tumor volume contour between DCTR U-Net as an artificial intelligence (AI) tool and oncologists. The red line represents the contour generated by artificial intelligence, while the blue line represents the contour depicted by oncologists.

### 4.1 Dataset

A total of 300 patients with NPC were diagnosed and histologically confirmed at Hainan Provincial People’s Hospital from June 2020 to December 2022, of whom 195 were male and 105 were female, aged 19–73 years, with a mean age of 42.56 years. The information of 300 nasopharyngeal cancer patients was extracted from their electronic medical records and reviewed by radiation oncologists with more than 10 years of experience. Finally, the actual nasopharyngeal cancer tumor boundaries were manually marked by physicians with more than 5 years of MRI imaging experience.

We divided 300 patients with nasopharyngeal cancer into 10 groups of 30 each on average, with 9 groups as the training set and 1 group as the validation set. To validate the effectiveness of our model, 10-fold cross-validation experiments were conducted. Based on our proposed model, we first built the U-Net base network model and then evaluated the performance of our U-Net combined with Dilated Residual Module and U-Net combined with Residual Transformer Module, and our proposed novel deep learning network model Dilated Convolution Transformer by 10-fold cross-validation Residual (DCTR U-Net) is compared with the mainstream network models to verify the effectiveness of our designed model.

### 4.2 Implementation and evaluation

In our 10-fold cross-validation experiments, we randomly divided all patients into 10 groups. One group was used as the validation set for each model, and the other nine groups were used as the training set. The network architecture was performed on PyTorch and trained for 600 epochs on two NVIDIA GeForce GTX 1080 Ti GPUs. The Adam optimizer was applied, setting β1 = 0.9 and β2 = 0.999, with an initial learning rate of 10^−4^, tuned to 10^−5^ after 400 training epochs. For the evaluation metrics, we use the computed dice similarity coefficient (DSC) and the average symmetric surface distance (ASSD) to assess the segmentation performance of all models. The value of DSC ranges from 0 to 1 means no spatial overlap; 1 means complete overlap and higher values indicate higher similarity (34). The DSC formula is defined as


(5) $$DSC=2\frac{A\cap B}{A+B}$$

where A and B denote the regions of the model segmentation result and the real segmentation result, respectively. $\text{A}\cap^{}\text{B}$ is the intersection region of A and B. The ASSD formula is defined as follows:


(6) $$ASSD=\frac{1}{\left|A\right|+\left|B\right|}(\sum_{a\in A}d\left(a,B\right)+\sum_{b\in B}d\left(b,A\right))$$

where A and B denote the set of pixel points on the surface of the model segmentation result and the real segmentation result, respectively. d(a, B) is the shortest Euclidean distance between the set of pixel points A and all points in B. d(b, A) is the shortest Euclidean distance between the set of pixel points B and all points in A. The smaller the ASSD is, the better the segmentation accuracy is.

## 5 Results

### 5.1 Ten-fold cross-validation experiment

**Figure 7:**
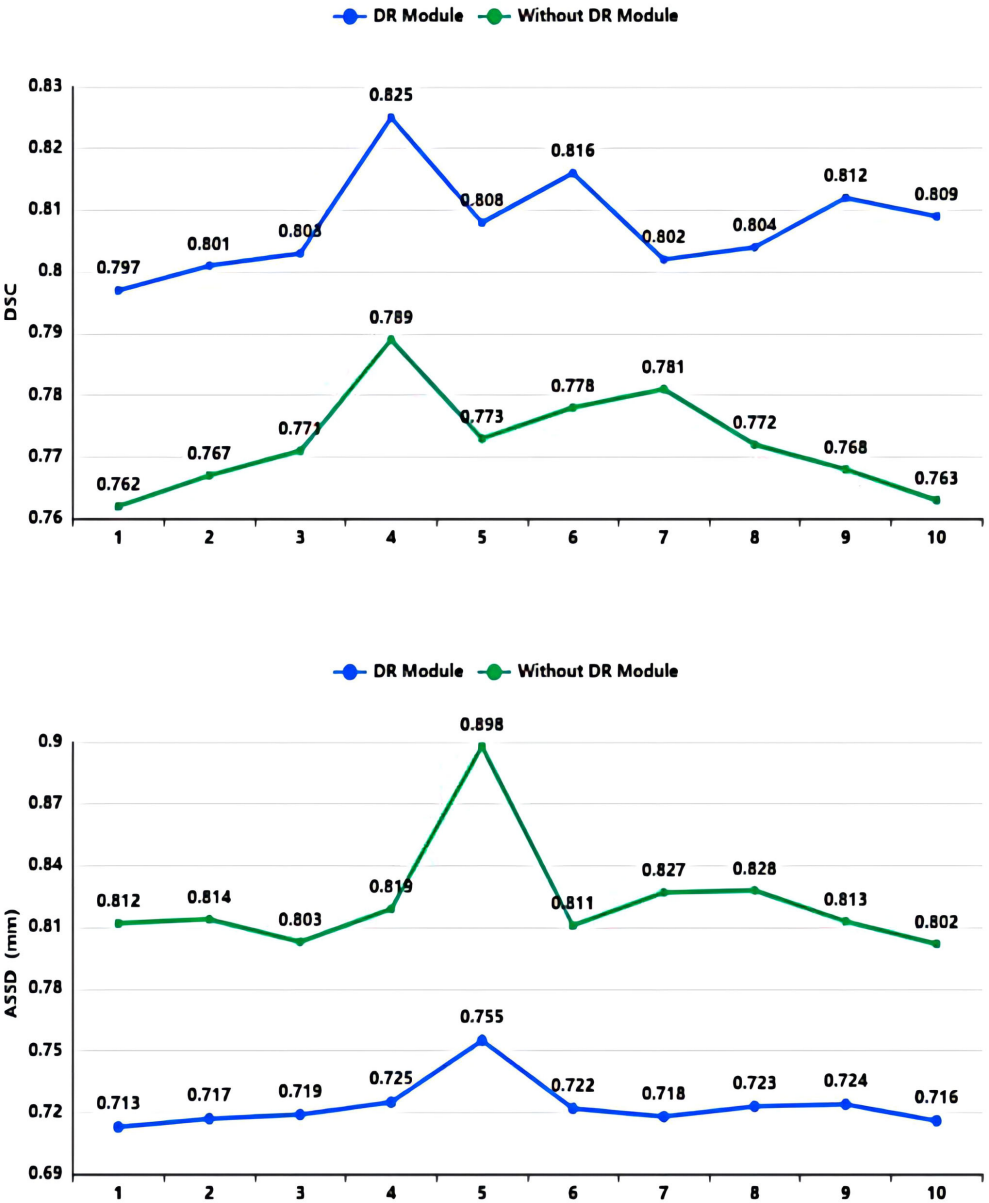
DSC value and ASSD value comparison diagram of Dilated Residual (DR) Module and without Dilated Residual (DR) Module.

As shown in **Table 2** and **Figure 7**, in the 10-fold cross-validation experiments, the average DSC and ASSD of the Dilated Residual Module are 0.808 and 0.723 mm, respectively. The average DSC and ASSD of the model without Dilated Residual Module are 0.772 and 0.823 mm, respectively. The results show that the model with the Dilated Residual Module has better performance than the model without the Dilated Residual Module, which indicates the effectiveness of the Dilated Residual Module.

**Table 2:** Average DSC and ASSD of each group of Dilated Residual (DR) Module and without Dilated Residual (DR) Module.

Model		1	2	3	4	5	6	7	8	9	10	Ave
DR Module	DSC	0.797	0.801	0.803	0.825	0.808	0.816	0.802	0.804	0.812	0.809	0.808
	ASSD (mm)	0.713	0.717	0.719	0.725	0.755	0.722	0.718	0.723	0.724	0.716	0.723
Without DR Module	DSC	0.762	0.767	0.771	0.789	0.773	0.778	0.781	0.772	0.768	0.763	0.772
	ASSD (mm)	0.812	0.814	0.803	0.819	0.898	0.811	0.827	0.828	0.813	0.802	0.823

**Figure 8:**
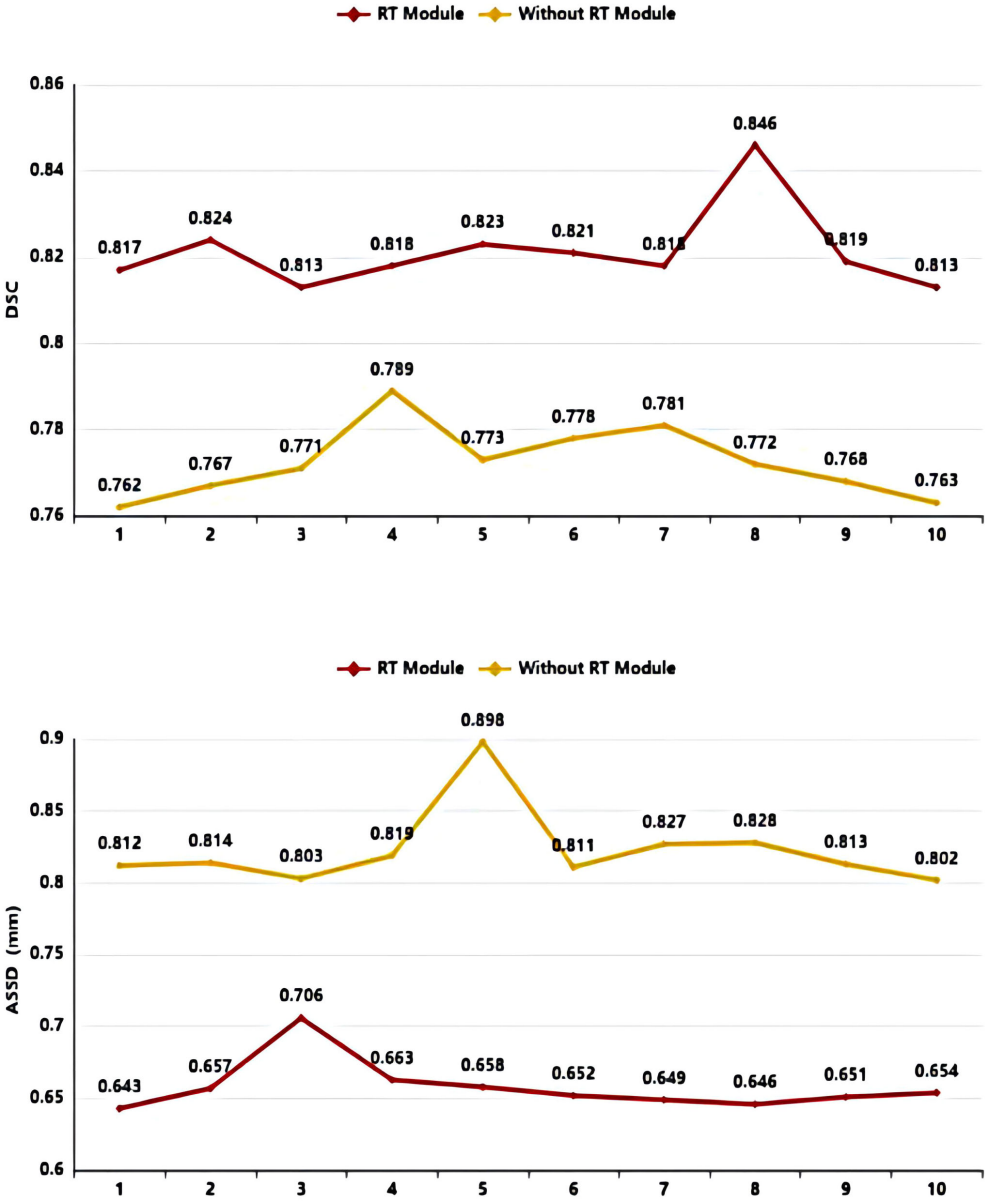
DSC value ASSD value comparison diagram of Residual Transformer (RT) Module and without Residual Transformer (RT) Module.

As shown in **Table 3** and **Figure 8**, in the 10-fold cross-validation experiments, the average DSC and ASSD of the Residual Transformer Module are 0.821 and 0.658 mm, respectively. The average DSC and ASSD of the model without the Residual Transformer Module are 0.772 and 0.823 mm, respectively. The results show that the model with the Residual Transformer Module has better performance than the model without the Residual Transformer Module, which indicates the effectiveness of the Residual Transformer Module.

**Table 3:** Average DSC and ASSD of each group of Residual Transformer (RT) module and without Residual Transformer (RT) module.

Model		1	2	3	4	5	6	7	8	9	10	Ave
RT Module	DSC	0.817	0.824	0.813	0.818	0.823	0.821	0.818	0.846	0.819	0.813	0.821
	ASSD (mm)	0.643	0.657	0.706	0.663	0.658	0.652	0.649	0.646	0.651	0.654	0.658
Without RT Module	DSC	0.762	0.767	0.771	0.789	0.773	0.778	0.781	0.772	0.768	0.763	0.772
	ASSD (mm)	0.812	0.814	0.803	0.819	0.898	0.811	0.827	0.828	0.813	0.802	0.823

### 5.2 Comparison of DCTR U-Net with mainstream models

The dilated convolution, transformer, and residual network structures are incorporated into the U-Net base model to form our network model DCTR U-Net, which is compared with the five currently dominant networks. The five dominant networks are as follows: U-Net (10), MultiResUNet (35), TransUNet (14), Swin-Unet (13), and UNETR (36).

**Figure 9:**
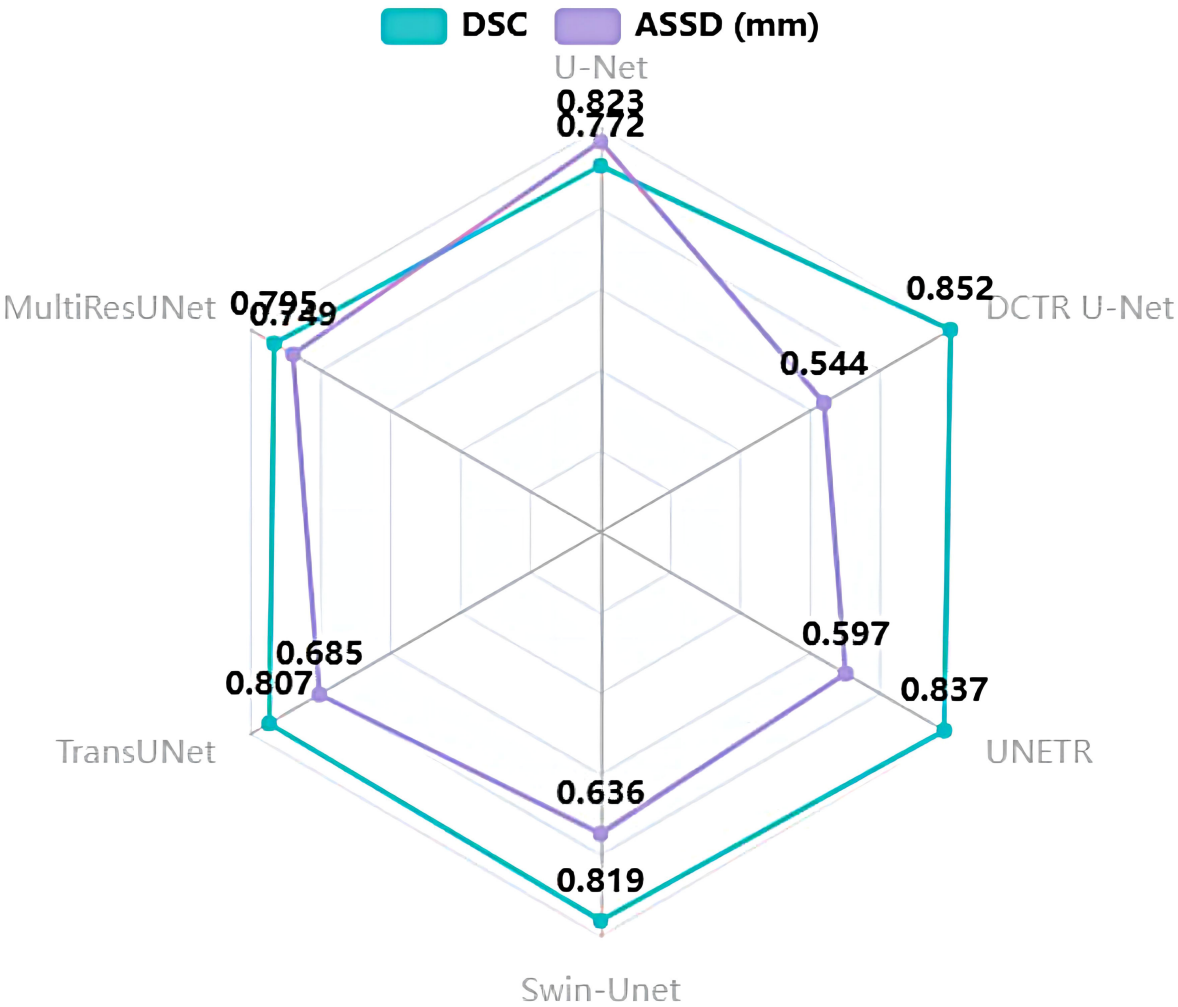
Radar diagram of DCTR U-Net and five mainstream networks.

All of the networks are processed using the same training datasets and validation datasets, and the quantitative analysis results are shown in **Table 4** and **Figure 9**. The experimental results show that our DCTR U-Net achieves the best results in both DSC and ASSD compared to the mainstream networks, where the DSC is 0.852 and the ASSD is 0.544 mm, and the image segmentation effect is greatly improved compared to other networks.

**Table 4:** Quantitative evaluation of DCTR U-Net with five mainstream networks for DSC and ASSD.

Network	DSC	ASSD (mm)
U-Net	0.772	0.823
MultiResUNet	0.795	0.749
TransUNet	0.807	0.685
Swin-Unet	0.819	0.636
UNETR	0.837	0.597
DCTR U-Net	0.852	0.544
Ground Truth	0.873	0.516

## 6 Discussion

In this paper, we proposed an automatic NPC segmentation network combining CNN and Transformer with the encoder–decoder structure of U-Net as the basic framework. To improve the accuracy of NPC segmentation and enhance the NPC segmentation precision, we added a Dilated Convolution Module, Transformer Module, and Residual Network Structure to the network for multi-scale extraction to supplement feature information and enhance the perceptual field to further improve the accuracy of the model. In experiments, our model has achieved better performance.

From a qualitative analysis perspective, as shown in **Figure 6**, compared to the tumor contour outlined by oncologists, DCTR U-Net has roughly segmented the overall contour of nasopharyngeal carcinoma, demonstrating good performance. From a quantitative analysis perspective, the DSC and ASSD values of DCTR U-Net are 0.852 and 0.544 mm, while the DSC and ASSD values of Ground Truth are 0.873 and 0.516 mm. Comprehensively consider ing the qualitative and quantitative analysis, we can conclude that there is almost no difference in indicators between these two methods of analysis. There is a high degree of consistency between the contours depicted by oncologists and those generated by the model.

As shown in **Table 2**, in our 10-fold cross-validation experiments with a total of 10 models, the models using our proposed Dilated Residual Module performed better. The Dilated Residual Module significantly improves the average DSC of groups 9 and 10, which is more than 0.04 higher than that of the model without Dilated Residual Module. Meanwhile, the other groups of models also achieved better results than the models without the Dilated Residual Module. Finally, we performed a quantitative analysis regarding the use or non-use of the Dilated Residual Module, where the values of the DSC were 0.808 and 0.772, and the values of the ASSD were 0.723 mm and 0.823 mm, respectively. Dilated Residual Module effectively improves the segmentation performance of NPC with an average DSC improvement of 0.036 and an ASSD reduction of 0.100 mm.

Similarly, as shown in **Table 3**, the model proposed by us, the Residual Transformer Module, performs better. It significantly improves the average DSC of groups 1, 2, 5, 8, 9, and 10, which is at least 0.050 higher than that of the model without the Residual Transformer Module. Meanwhile, the other groups of models also achieved better results than the models without the Dilated Residual Module. Finally, we performed a quantitative analysis regarding the use or non-use of the Residual Transformer Module, where the values of the DSC were 0.821 and 0.772, and the values of the ASSD were 0.658 mm and 0.823 mm, respectively. The Residual Transformer Module proposed by us effectively improved the segmentation performance of the NPC with an average DSC improvement of 0.049 and the ASSD was reduced by 0.165 mm.

### 6.1 Ablation experiment

**Figure 10:**
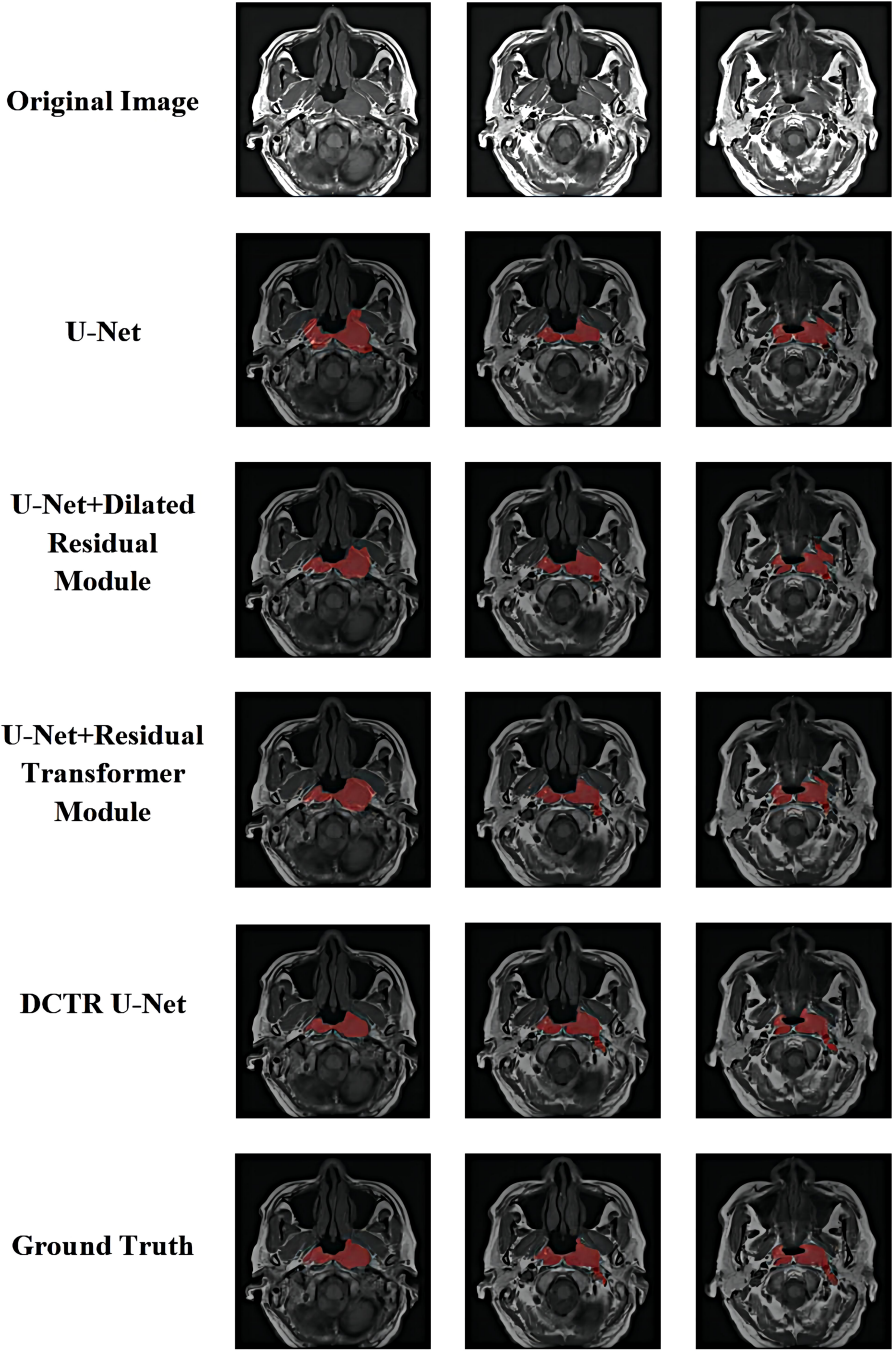
Visualization and qualitative comparison results of ablation experiments.

**Figure 10** shows the qualitative comparison results of ablation experiments. For each subplot, the first row as the comparison result shows the original image, the second row indicates the U-Net segmentation result, the third row indicates the U-Net + Dilated Residual module segmentation result, the fourth row indicates the U-Net + Residual Transformer Module segmentation result, the fifth row indicates the DCTR U-Net segmentation result, and the sixth row indicates the actual physician labeled correct result.

**Table 5** presents the comparison results under different modules that have validated the effectiveness of the Dilated Residual Module and the Residual Transformer Module. We used the first data set for training in a 10-fold cross-validation experiment, controlling all training parameters in the same way. When only using U-Net, we obtained an average DSC of 0.772 and an ASSD of 0.823 mm. The U-Net is used as the base architecture and combines the Dilated Residual Module and the Residual Transformer Module respectively, as well as both of them, to conduct experimental comparison and quantitative analysis. When combining the U-Net and the Dilated Residual Module alone, the average DSC value and average ASSD value are 0.808 and 0.723 mm. Its average DSC value is 0.036 higher than when using U-Net architecture alone. Its average ASSD value is 0.100 mm lower than when using U-Net architecture alone. Meanwhile, when combining the U-Net and the Residual Transformer Module alone, the average DSC value and average ASSD value are 0.821 and 0.658 mm. Its average DSC value is 0.049 higher than when using U-Net architecture alone, and its average ASSD value is 0.165 mm lower than when using U-Net architecture alone. Also, the performance is significantly improved and is similar to that of the Dilated Residual Module alone. However, when U-Net uses both the Dilated Residual Module and the Residual Transformer Module, the results show that the performance is better than when either module is used alone, with the average DSC and ASSD reaching 0.852 and 0.544 mm, respectively. The model with the combination of the Dilated Residual Module and the Residual Transformer Module obtains the best results, which is more stable than the other models. From the results of the experiments, we concluded the following.

**Table 5:** Quantitative analysis results of ablation experiments.

Model	DSC	ASSD (mm)
U-Net	0.772	0.823
U-Net+Dilated Residual Module	0.808	0.723
U-Net+Residual Transformer Module	0.821	0.658
DCTR U-Net	0.852	0.544

On the one hand, using the Dilated Residual Module and the Residual Transformer Module respectively can improve the performance of the model, and the progress of NPC segmentation can be further improved when used simultaneously. On the other hand, the performance improvement of the U-Net model is obvious by using the Dilated Residual Module alone, and the performance improvement is also good by using the Residual Transformer Module alone. Thus, they demonstrated the importance and necessity of the Dilated Residual Module and the Residual Transformer Module for the improvement of the U-Net model.

A total of 300 patients were trained and evaluated in experiments to ensure that results are reliable. Our model DCTR U-Net is compared with current mainstream models and the experimental results are shown in **Table 3**. DCTR U-Net showed the best experimental results on DSC and ASSD. However, our study has the following limitations.

### 6.2 Limitations of the experiment

Owing to the GPU memory limitation and keeping the original image without compression, we finally set the batch size to 6. A larger batch size can help improve the performance. Using Group Normalization (37), one can solve this problem and improve accuracy. In the 10-fold cross-validation experiment, we trained a total of 30 models in the residual module model and the base model and set the epoch to 600 to obtain the training results in order to reduce the training time. The performance of the model can be improved if the epoch is set larger.

### 6.3 Prospects

In this study, we proposed and evaluated a novel U-Net-based automatic segmentation network for MR images of NPC. Although U-Net has been widely used in tumor segmentation, the DCTR U-Net network structure proposed by us achieves more reliable and better segmentation results than others. Combining the Dilated Residual Module and the Residual Transformer Module successfully improves the NPC tumor segmentation performance. Tenfold cross-validation results show that the method has better recognition for the Dilated Residual Module and the Residual Transformer Module. The future research direction is to optimize the infrastructure of the DCTR U-Net network and compensate for the impact of insufficient data on experiments by using semi-supervised learning methods.

## 7 Conclusion

We proposed a new deep learning network model, Dilated Convolution Transformer Residual (DCTR U-Net), based on the U-Net network, combining the Dilated Convolution, Transformer, and Residual Network Structures, and when using the same dataset under the same test conditions, both the proposed Dilated Residual Module and the Residual Transformer Module perform better than the network using only image information under the same test conditions. After the last two modules are reasonably spliced, the unique residual module combining the dilated convolution, transformer, and residual structures can effectively perform multi-scale extraction, complement feature information, and enhance the perceptual field to improve the results, making the NPC segmentation process more accurate and stable, and significantly improving the performance of automatic NPC tumor sketching.

## Data availability statement

The data sets used and analyzed in this study are available from the corresponding authors upon reasonable request. However, the data came from the Hainan Provincial People’s Hospital and only a small portion of the data can be disclosed after communication; please contact the corresponding author for more details. Requests to access the datasets should be directed to Chinabasketball715@163.com.

## Ethics statement

The studies involving human participants were reviewed and approved by Hainan Provincial People’s Hospital ethics committee. The patients/participants provided their written informed consent to participate in this study. Written informed consent was obtained from the individual(s) for the publication of any potentially identifiable images or data included in this article.

## Author contributions

Conceptualization, YZ and CS; methodology, PZ; software, SS and KZ; validation, WL; formal analysis, JL; investigation, ZZ. All authors contributed to the article and approved the submitted version.
